# Intravenous thrombolysis for acute ischemic stroke due to vertebral artery dissection: A case report

**DOI:** 10.1097/MD.0000000000045581

**Published:** 2025-10-24

**Authors:** Han Luo, Shanshan Li, Xiongbin Cao, Bo Liu

**Affiliations:** a Department of Neurology, Shenzhen Longhua District Central Hospital, Shenzhen, Guangdong Province, PR China.

**Keywords:** acute ischemic stroke, intravenous thrombolysis, isolated vertigo, posterior inferior cerebellar artery, vertebral artery dissection

## Abstract

**Rationale:**

Vertebral artery dissection (VAD) is a frequently overlooked cause of posterior circulation stroke in young adults, and the safety of intravenous thrombolysis in this setting remains debated. We report a case of acute ischemic stroke secondary to V3-segment VAD that progressed despite dual antiplatelet therapy, responded promptly to recombinant tissue-plasminogen activator thrombolysis, and showed complete vascular healing at 3 months. The study aim is to underscore that VAD is not an absolute contraindication to thrombolysis and to highlight the value of early recognition and individualized thrombolytic therapy for improving outcomes in young patients with VAD-related stroke.

**Patient concerns:**

The patient, a 30-year-old male, was admitted to the hospital at 11:45 on January 24, 2019, due to transient episodic weakness in the right limbs that occurred 2 hours ago, lasting for 30 minutes.

**Diagnoses:**

Left vertebral artery V3 dissection; bulbar lacunar infarction; grade 2 hypertension; polycystic kidney; polycystic liver; lung infection.

**Interventions:**

The patient was diagnosed with a dissection of the V3 segment of the vertebral artery through cranial computed tomography angiography. The disease continued to progress despite dual antiplatelet aggregation therapy with aspirin and clopidogrel. Therefore, intravenous thrombolytic therapy was administered.

**Outcomes:**

On February 11, 2019, the patient was discharged after an improvement in symptoms. There were no neurological deficit symptoms observed at that time. The patient did not experience any subsequent acute ischemic stroke events. The patient showed no hemorrhagic transformation or serious complications. Three months later, the patient had returned to work. Follow-up imaging after 3 months showed complete repair of the vessel with vertebral artery dissection.

**Lessons:**

The most common clinical manifestations of VAD include dizziness/vertigo, headache, neck pain, and secondary neurologic deficits. However, these symptoms are often nonspecific. This can lead to frequent misdiagnosis or missed diagnosis of the disease. The efficacy and safety of intravenous thrombolysis for ischemic stroke due to VAD remains controversial due to its potential risks. VAD is not considered a contraindication to intravenous thrombolysis.In some cases, VAD endometrial damage can be repaired on its own after regular medical treatment.

## 1. Introduction

Vertebral artery dissection (VAD) is characterized by the formation of an intramural hematoma caused by vertebral artery intimal injury and tear. This allows blood to flow into and separate the layers of the blood vessel wall. This process can lead to blood vessel narrowing, occlusion, or the formation of a dissecting aneurysm.^[1]^ VAD is a common cause of ischemic stroke in young adults. It has a complex pathogenesis and is often related to factors such as trauma (including neck massage, sneezing, severe coughing, physical activity, etc), fibromuscular dystrophy, genetic factors, and hypertension.^[1]^ Due to the specific anatomical features and hemodynamic changes of the vertebral arteries, VAD predisposes to the V3 and V4 segments of the vertebral arteries.^[2]^ Approximately 63% of patients with VAD develop acute ischemic stroke (AIS), with a higher proportion of VAD-related AIS (66%) occurring in the extracranial segments and only 32% in the intracranial segment.^[3]^ VAD-related AIS is commonly caused by perforating artery occlusion, arterial–arterial embolism. Patients receiving thrombolytic therapy may experience some degree of neurological improvement in the short term. However, the long-term prognosis for many cases shows limited significant improvement, particularly regarding the impact of thrombus dissolution in the false lumen on blood flow restoration.^[4]^ The lack of robust support from large-scale randomized controlled trials poses substantial challenges for clinicians when selecting treatment strategies. This article introduces the diagnosis and treatment process of a patient with acute ischemic stroke related to vertebral artery dissection. It also aims to enhance the accuracy of VAD diagnosis and promote more effective interventions.

## 2. Case presentation

### 2.1. Clinical presentation

The patient, a 30-year-old male, was admitted to the hospital at 11:45 on January 24, 2019, due to transient episodic weakness in the right limbs that occured 2 hours ago, lasting for 30 minutes. The patient suddenly developed right limb weakness while turning his head at work 2 hours ago, which persisted for about 30 minutes before returning to normal. During the course of the episode, he occasionally felt dizzy. There were no recurrent attacks, no amaurosis, no visual rotation, no double vision, no slurred speech, no choking while drinking water, no difficulty swallowing, and no nausea or vomiting. History of hypertension with blood pressure up to 135/95 mm Hg, managed with oral administration of valsartan capsules, resulting in acceptable blood pressure control. History of polycystic kidney disease. Denial of chronic medical history such as diabetes and coronary heart disease. No unhealthy living habits like smoking or drinking. He denied any family history of strokes, and he had healthy, nonconsanguineous parents. Temperature: 36.5°C, pulse: 84 beats/min, respiratory rate: 20 beats/min, blood pressure: 148/104 mm Hg. Breath sounds in both lungs were coarse, with no obvious rhonchi or moist rales heard. Heart rate was 84 beats per minute with a uniform rhythm. No obvious murmur was heard in the auscultation area of each valve. The abdomen was flat and soft, with no obvious tenderness or rebound tenderness in the whole abdomen. Specialist examination: The patient was fully conscious and spoke clearly. Eye movements were normal in all directions, with no diplopia or nystagmus. Pupils were equal, measuring 2.5 mm, and reactive to light. Bilateral frontal lines were symmetrical, with good binocular closure force. Bilateral nasolabial folds were symmetrical, and mouth corners were not crooked. Hearing was normal, with no choking while drinking water or difficulty swallowing. The soft palate mobility was symmetrical, with a normal gag reflex. Neck rotation and shoulder shrug were symmetrical and strong bilaterally. Tongue protrusion was midline, with no tongue muscle atrophy. Limb muscle strength was grade 5, and muscle tone was normal. Limb tendon reflexes were normal, and Babinski signs were negative bilaterally. The bilateral finger-to-nose test and heel-to-shin test were normal. Deep and superficial sensations were normal. The neck was soft, and both Kernig and Brudzinski signs were negative.

### 2.2. Assistant examinations

**Figure 1.**
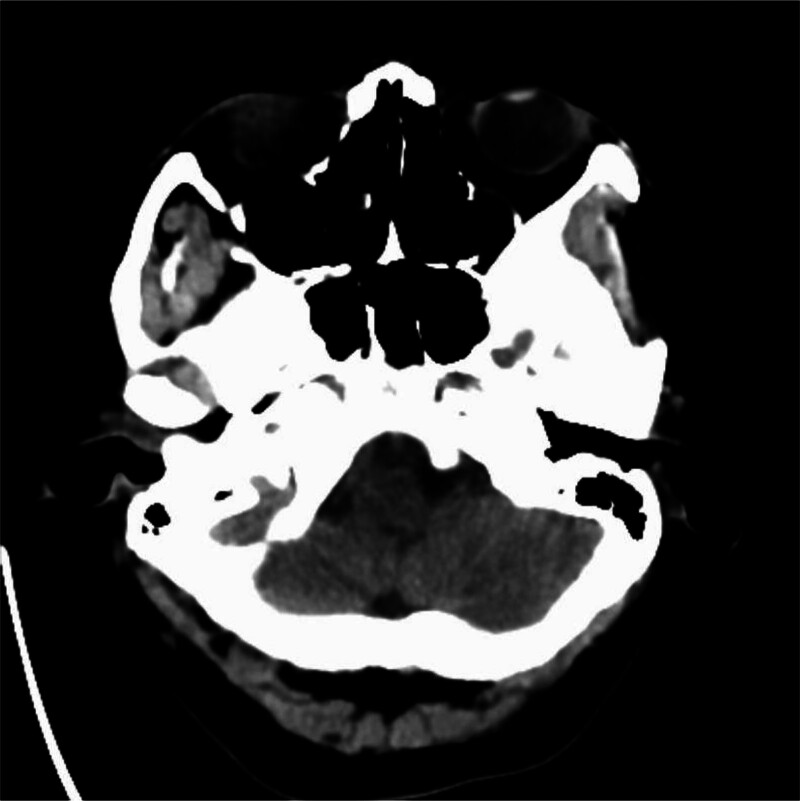
On January 24, 2019, non-contrast CT images of the patient.

**Figure 2.**
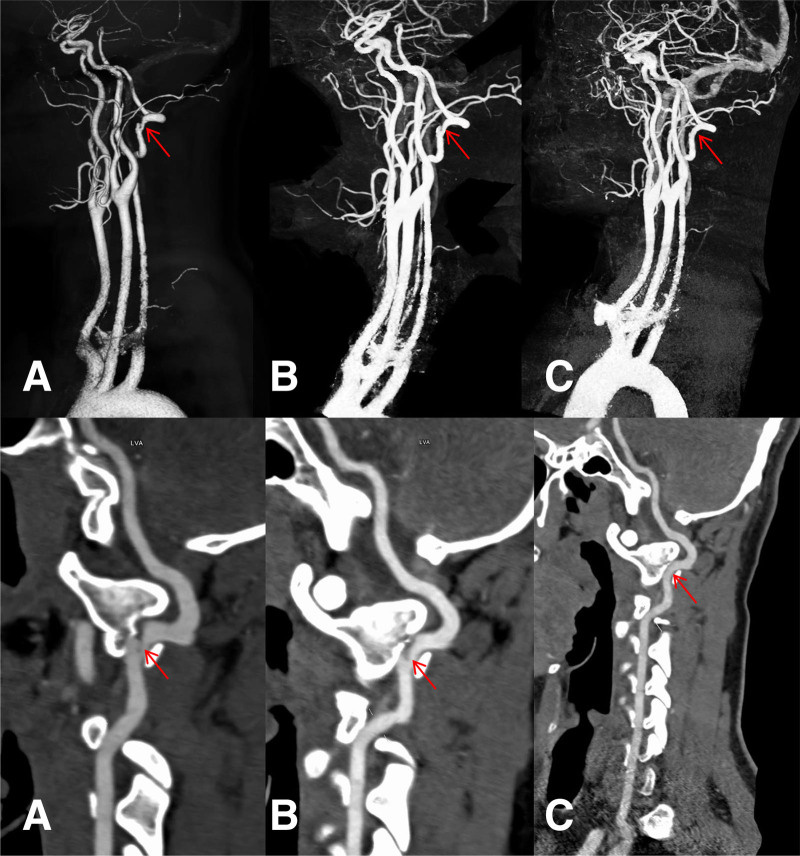
Images of cranial CTA at different times (arrows indicate anatomical location). (A) On January 24, 2019, CTA of the head and neck revealed moderate to severe stenosis and dissecting aneurysm formation in the lumen of the V3 segment of the left vertebral artery. (B) On October 1, 2019, CTA of the head and neck showed moderate to severe stenosis and dissecting aneurysm formation in the lumen of the V3 segment of the left vertebral artery. (C) On January 24, 2019, CTA of the head and neck showed complete repair of the left vertebral artery, with near-complete regression of the dissecting aneurysm. CTA = computed tomography angiography.

**Figure 3.**
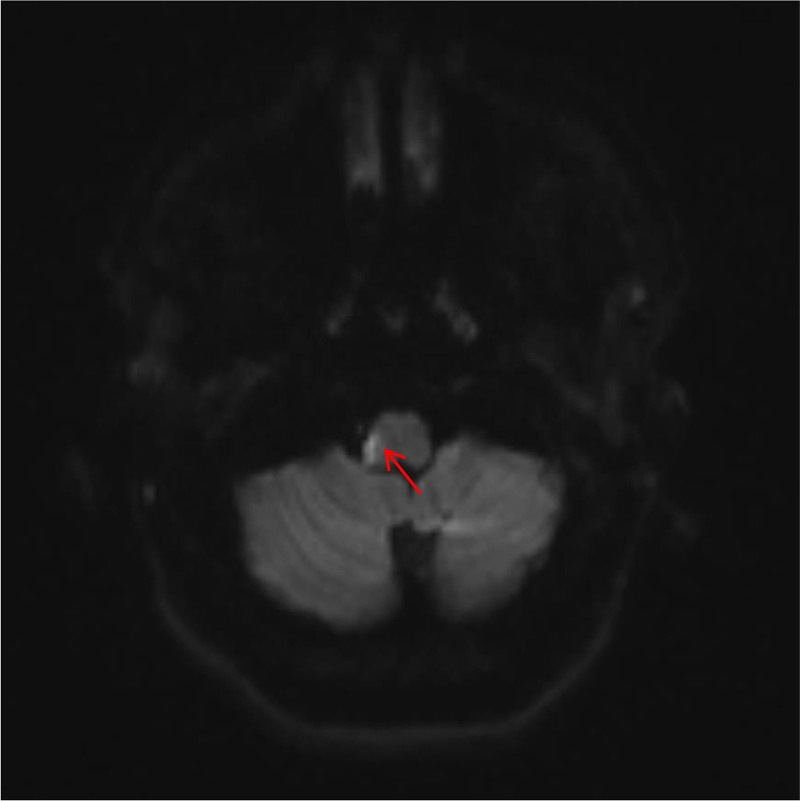
On January 25, 2019, the DWI sequence suggests a lacunar infarction in the medulla oblongata (arrows indicate anatomical location). DWI = diffusion weighted imaging.

Blood lipids: Low-density lipoprotein cholesterol was 3.39 mmol/L. There were no obvious abnormalities in fasting blood glucose, hypersensitive C-reactive protein, thyroid function, glycosylated hemoglobin, urinalysis, or stool routine examination. Cranial computed tomography (CT) (January 24, 2019): No obvious abnormalities were observed in the transverse sections of the cranial non-contrast CT scan (Fig. 1). Computed tomography angiography (CTA) of the head and neck (January 24, 2019): Dissection of the V3 segment of the left vertebral artery and a slender right vertebral artery (Fig. 2A). Magnetic resonance imaging of the brain: Bulbar lacunar infarction was identified (Fig. 3). Cranial transcranial Doppler: No definite blood flow signal was detected in the right vertebral artery. Blood flow spectrum and blood flow velocity in the left vertebral artery were normal. Carotid + vertebral artery ultrasound: There was no significant stenosis or occlusion of bilateral carotid arteries. The right vertebral artery showed slow blood flow velocity with high resistance. The flow spectrum and blood flow velocity in the left vertebral artery were normal. Cardiac ultrasound: EF: 71%, no abnormalities in cardiac morphology and structure. Normal left ventricular systolic and diastolic function. Abdominal ultrasound: Polycystic liver. Urinary ultrasound: Polycystic kidney. Chest CT: Dorsal and posterior basal segment infections in the inferior lobes of bilateral lungs.

### 2.3. Final diagnosis

Left vertebral artery V3 dissection; bulbar lacunar infarction; grade 2 hypertension; polycystic kidney; polycystic liver; lung infection.

### 2.4. Interventions

After admission, the patient was started on dual antiplatelet aggregation and antihypertensive therapy with aspirin and clopidogrel, along with other treatments. At 22:00 on the day of admission, the patient developed dizziness, slurred speech, choking while drinking water, and difficulty swallowing. Physical examination revealed unclear anarthria and pseudobulbar palsy. These symptoms persisted for more than 30 minutes without improvement. A clinical diagnosis of posterior circulation cerebral infarction was confirmed. Recombinant tissue-plasminogen activator (rt-PA) thrombolytic therapy was administered at 22:35. Twenty-four hours after thrombolysis, dual antiplatelet aggregation therapy with aspirin and clopidogrel was administered.

### 2.5. Outcome and follow-up

On February 10, 2019, a CTA of the head and neck was reviewed. It revealed moderate to severe stenosis and dissecting aneurysm formation in the lumen of the V3 segment of the left vertebral artery (at the atlas intervertebral foramina plane), as well as a slender right vertebral artery (Fig. 2B). On February 11, 2019, the patient was discharged after an improvement in symptoms. The patient’s symptoms of dizziness, slurred speech, choking while drinking water, and difficulty swallowing had resolved. There were no neurological deficit symptoms observed at that time, with both the National Institutes of Health Stroke Scale and modified Rankin scale scores at 0. On April 24, 2019, the patient had experienced no recurrence of transient ischemic attack (TIA) or cerebral infarction. He showed no hemorrhagic transformation or serious complications. He had no neurological deficit symptoms. The patient had returned to work, with an modified Rankin scale score of 0. A follow-up head and neck CTA showed a slender right vertebral artery. There were no significant abnormalities in the other arteries (Fig. 2C).

## 3. Discussion

### 3.1. The anatomy of the vertebral arteries and their variations are complex

The vertebral artery is divided into 4 segments: the V1 segment (pre-foraminal segment), the V2 segment (foraminal segment), the V3 segment (atlantoaxial segment), and the V4 segment (intracranial segment). The first 3 segments are extracranial, while the fourth segment is intracranial.^[5]^ Due to the wide range of motion of the cervical spine, congenital anatomical variations of the atlantoaxial vertebrae and vertebral artery, and occipitalization of the atlantoaxial vertebrae, the V3 segment is susceptible to minor mechanical injuries, such as those caused by neck massage. The V4 segment is a tortuous vessel susceptible to hemodynamic changes. This makes VAD more likely to occur in the V3 and V4 segments of the vertebral artery.^[2]^ With advancements in imaging technology, anatomical variations of the vertebral artery have gradually been emphasized. These variations include differences in origin, diameter, course, and branching, as well as fenestration and duplication malformations.^[6]^ Morphological variations in the vertebral arteries not only affect hemodynamics, flow reserve, and compensatory capacity but may also significantly increase the risk of VAD or posterior circulation AIS.^[7,8]^ In the general population, about 23.3% of bilateral vertebral arteries are roughly equal in diameter, 30% show dominance of the right vertebral artery, and 46.6% show dominance of the left vertebral artery.^[6]^ Patients with unilateral vertebral artery dysplasia have a limited compensatory capacity in the contralateral vertebral artery. When VAD occurs, it is more likely to result in insufficient blood supply to branches of the vertebral or basilar arteries, particularly the posterior inferior cerebellar artery (PICA). This can lead to the development of AIS.^[8]^ The patient’s right vertebral artery was slender. The dominant vertebral artery on the left side formed a dissection after head rotation. The patient experienced a TIA, and symptoms worsened on the night of admission. These events are considered to be partially related to the patient’s congenital vascular variation. The etiology of cerebral infarction in this patient is more likely arterial embolism caused by the dissection, as a more severe headache would occur if the V3 dissection extended to the basilar artery. However, transcranial Doppler microemboli monitoring and other approaches have limited value in determining the etiology. Therefore, patients presenting with symptoms of posterior circulation ischemia should undergo early screening with CTA or MRA of the vertebral arteries.

### 3.2. The clinical manifestations of VAD are atypical

The most common clinical manifestations of VAD include dizziness/vertigo (58%), headache (51%), neck pain (46%), and secondary neurologic deficits.^[3]^ However, these symptoms are often nonspecific. This can lead to frequent misdiagnosis or missed diagnosis of the disease. Vertebrobasilar insufficiency syndrome, caused by dynamic compression of the vertebral arteries due to head rotation or specific body positioning. This condition, known as Bow Hunter syndrome, is a common cause of vertebral artery V3 dissection.^[9]^ TIAs or subarachnoid hemorrhages associated with VAD are relatively rare.^[3]^ One contributing factor is that VAD often leads to ischemia in the medial branch of PICA, presenting mainly as isolated vertigo. This symptom is easily mistaken for vestibular neuritis, which can delay the timely diagnosis of TIA and result in cerebral infarction. It should also be noted that some patients with atypical acute coronary syndrome may present with neck, shoulder, and posterior occipital pain accompanied by throat obstruction, without the typical symptoms of chest tightness or chest pain. These symptoms closely resemble those of VAD, making differential diagnosis more challenging. CT has certain limitations in the evaluation of VAD-related AIS, particularly in detecting branch infarcts of the vertebral or basilar arteries caused by dissection. In contrast, magnetic resonance imaging is able to clearly reveal the fine lesions of VAD-related cerebral infarction. The patient presented with TIA on admission. These symptoms were triggered by head rotation. Therefore, we performed a CTA of the head and neck to confirm the presence of VAD. We also administered aspirin and clopidogrel as dual antiplatelet aggregation therapy. On the night of admission, the patient developed dizziness, slurred speech, choking while drinking water, and difficulty swallowing. These symptoms persisted for more than 30 minutes without improvement. A clinical diagnosis of posterior circulation AIS was confirmed, suspected to be caused by microembolism of the PICA branch due to dissection. As a result, intravenous thrombolytic therapy was administered.

### 3.3. Intravenous thrombolytic therapy for VAD needs to be individualized

The efficacy and safety of intravenous thrombolysis in the treatment of VAD-related ischemic stroke remain controversial. The mechanism of ischemic stroke due to VAD may result from perforating artery occlusion, arterial-arterial embolism, or hypoperfusion due to hemodynamic changes.^[10]^ Intravenous thrombolysis is generally effective for ischemic stroke treatment. However, when VAD leads to ischemic stroke, the dissection may be accompanied by arterial wall damage and sub-endovascular hematoma formation. Intravenous thrombolysis may exacerbate vascular injury and increase the potential risk of bleeding.^[1]^ However, intravenous thrombolytic therapy for ischemic stroke caused by neck dissection has been shown to be as effective as it is in the treatment for ischemic stroke due to other reasons. In addition, this therapy does not increase the risk of symptomatic intracranial hemorrhage.^[1,11]^ The patient was treated with aspirin and clopidogrel dual antiplatelet aggregation therapy, but it failed to prevent disease progression. Subsequently, the patient experienced choking while drinking water and difficulty swallowing. We were unable to determine whether the disease progression was due to involvement of the same perforating blood supply area responsible for the initial symptoms or embolism in a different perforating blood supply area. Therefore, rt-PA intravenous thrombolytic therapy was administered to the patient, resulting in a good prognosis. However, there were deficiencies in our treatment. After successful thrombolysis, the patient continued on aspirin and clopidogrel dual antiplatelet aggregation therapy. Due to the condition limitation at that time, the patient was not evaluated in time for the possibility of clopidogrel resistance. If the patient’s symptoms remain uncontrolled despite regular medical treatment, it is still important to consider whether there is an indication for early endovascular intervention. Therefore, in clinical decision-making, we need to fully weigh the efficacy and safety of intravenous thrombolytic therapy for VAD and formulate individualized treatment strategies.

### 3.4. Influencing factors of vascular repair in the later stages of VAD

The repair time for intimal injury in most VADs ranges from 3 to 6 months. In rare cases, it can be extended up to 12 months. Residual stenosis or occlusion is usually not significantly associated with a recurrent AIS event within 6 months after dissection.^[1]^ However, this affects the duration of antiplatelet aggregation/anticoagulation therapy in the later stages. The basilar artery, formed by the convergence of the bilateral vertebral arteries at the inferior border of the pons, is the backbone of the posterior circulation. Therefore, although dissection impairs blood flow in 1 vertebral artery, the prognosis of most patients with VAD is good. Mortality and recurrence rates are low, with an MRS score of 0 to 1 in approximately 67% of follow-up patients.^[3]^ The main influencing factors of VAD endovascular repair are sex, smoking, the presence or absence of branch involvement of the vertebral artery, and the angle of confluence of the vertebral and basilar arteries. Generally, VAD vessels are more difficult to repair in patients with PICA involvement and smaller angles of confluence of the vertebral and basilar arteries.^[10]^ At long-term follow-up, about 5% to 40% of carotid artery dissection aneurysms resolve spontaneously, 15% to 30% shrink, and 50% to 60% remain unchanged. VAD aneurysms have a higher likelihood of repair compared to carotid artery dissection aneurysms.^[12]^ In this patient’s case, a 3-month follow-up CTA of the head and neck showed significant repair of the VAD intimal damage and spontaneous resolution of the dissecting aneurysm, indicating a good prognosis. However, the patient only underwent CTA reexamination at 3 months. The absence of serial imaging during the acute phase (within 7 days) and long-term follow-up (6–12 months) makes it impossible to dynamically delineate the dissection repair process.

## 4. Conclusions

VAD often presents clinically with dizziness/vertigo, headache, neck pain, and secondary neurologic deficits. However, these symptoms are often nonspecific. This can lead to frequent misdiagnosis or missed diagnosis of the disease. Due to the wide range of motion of the cervical spine, congenital anatomical variations of the atlantoaxial vertebrae and vertebral artery, and occipitalization of the atlantoaxial vertebrae, the V3 segment is susceptible to minor mechanical injuries. These injuries, such as those caused by neck massage, can result in vertebral artery dissection or Bow Hunter syndrome. Aspirin and clopidogrel dual antiplatelet aggregation therapy is one of the options for the medical treatment of VAD. While the efficacy and safety of intravenous thrombolysis in the treatment of VAD-related ischemic stroke remain controversial, VAD is not considered a contraindication to intravenous thrombolysis. If a patient’s symptoms are not effectively controlled after regular medical treatment, it is still necessary to consider whether there is an indication for early endovascular intervention. In some cases, VAD endometrial damage can be repaired on its own after regular medical treatment.This is a 1-patient report without a control group. Consequently, the magnitude of benefit or hemorrhage risk attributable to rt-PA in VAD stroke cannot be quantified and generalizability is limited. Multicentre, prospective cohorts or registries are needed to compare the efficacy and safety of thrombolysis with other antithrombotic or interventional approaches in VAD-related stroke.

## Acknowledgments

We would like to express our gratitude to the patient’s family members for granting permission to use the patient’s clinical data in this paper and for the publication of this research.

## Author contributions

**Conceptualization:** Han Luo.

**Data curation:** Han Luo, Shanshan Li, Xiongbin Cao.

**Formal analysis:** Han Luo, Xiongbin Cao.

**Funding acquisition:** Bo Liu.

**Methodology:** Xiongbin Cao.

**Resources:** Xiongbin Cao.

**Software:** Bo Liu.

**Supervision:** Bo Liu.

**Validation:** Bo Liu.

**Writing – original draft:** Han Luo.

**Writing – review & editing:** Bo Liu.
